# QuEChERS结合超高效液相色谱-串联质谱法同时测定畜禽肉中44种食源性兴奋剂和6种孕激素

**DOI:** 10.3724/SP.J.1123.2021.12005

**Published:** 2022-05-08

**Authors:** Yuechao FENG, Jianfeng WANG, Fan HOU, Qi DING, Hongyu CHU, Yan LIU

**Affiliations:** 北京市科学技术研究院分析测试研究所(北京市理化分析测试中心), 北京市食品安全分析测试工程技术研究中心, 北京 100089; Institute of Analysis and Testing, Beijing Academy of Science and Technology (Beijing Center for Physical & Chemical Analysis), Beijing Engineering Research Center of Food Safety Analysis, Beijing 100089, China

**Keywords:** QuEChERS, 超高效液相色谱-串联质谱, 食源性兴奋剂, 孕激素, 畜禽肉, QuEChERS, ultra-performance liquid chromatography-tandem mass spectrometry (UPLC-MS/MS), foodborne stimulants, progestogens, meat

## Abstract

利用QuEChERS结合超高效液相色谱-串联质谱法建立了畜禽肉中44种兴奋剂和6种孕激素的检测技术。样品粉碎均质后加入内标,依次加入水和含0.5%乙酸的乙腈溶液振荡提取后,加入氯化钠和无水硫酸镁脱水离心,上清液采用PSA、C_18_、中性氧化铝和无水硫酸镁分散固相萃取材料进行净化,净化液经氮气吹干,复溶后用超高效液相色谱-串联质谱仪测定。被测物采用ACQUITY BEH C_18_(100 mm×2.1 mm, 1.7 μm)色谱柱,以0.1%甲酸-5 mmol/L乙酸铵水溶液和甲醇为流动相分离,在电喷雾正离子模式下,以多反应监测(MRM)方式采集,采用基质匹配标准曲线内标法定量分析。50个被测物包含6类化合物:*β*_2_-激动剂类(19个)、*β*-阻断剂类(3个)、蛋白同化激素类(11个)、糖皮质激素类(8个)、利尿剂类(3个)、孕激素(6个)。所有被测物在各自的范围内线性关系良好,相关系数>0.99, *β*_2_-激动剂类和*β*-阻断剂类的线性范围为0.1~20 μg/L,糖皮质激素类的线性范围为0.5~200 μg/L,蛋白同化激素类、孕激素类、利尿剂类的线性范围为0.2~50 μg/L。方法的定量限范围为0.1~0.4 μg/kg。在低、中、高3个浓度水平下的加标回收率试验中,50种目标化合物在鸡肉、猪肉、牛肉、羊肉中的平均回收率范围为50.3%~119.9%,相对标准偏差(RSD, *n*=6)范围为0.42%~15.1%。采用该法和国标方法(GB/T 21981-2008)同时对市售的9个肉样品(包括3个牛肉、3个猪肉、2个鸡肉、1个鸭肉)进行了比对检测,对检出的氢化可的松、可的松含量采用*t*检验进行统计学分析,结果表明,两种方法测得的数据没有显著性差异。采用该法测定了12个来自某养殖场的牛肉样品,结果共检出4种食源性兴奋剂,其中氢化可的松的含量范围为3.3~22.6 μg/kg,检出率为100%;可的松的含量范围为1.5~2.1 μg/kg,检出率为67%;雄烯二酮含量范围为0.7~1.2 μg/kg,检出率为17%;睾酮含量范围为0.6~1.5 μg/kg,检出率为42%。该法操作简单,准确灵敏,重复性好,适用于不同种类畜禽肉中食源性兴奋剂和孕激素的检测。

## Abstract (en)

To ensure the success of large-scale sporting events, prevent the contamination of food by prohibited substances, and evaluate the risk of foodborne stimulants and other hormones in food, it is necessary to establish a high-throughput, rapid, and accurate detection method for foodborne stimulants and other hormones. In this study, a QuEChERS method is proposed for the simultaneous determination of 44 foodborne stimulants and 6 progestogens using ultra-performance liquid chromatography-tandem mass spectrometry (UPLC-MS/MS). The analyzed foodborne stimulants include 19 *β*_2_-agonists, 3 *β*-blockers, 11 anabolic agents, 8 glucocorticoids, and 3 diuretics. A meat sample was crushed and homogenized, following which the internal standard was added. Subsequently, the sample was shaken and extracted with water and an acetonitrile solution containing 0.5% acetic acid, then dehydrated and centrifuged with sodium chloride and anhydrous magnesium sulfate. The supernatant was purified by PSA, C_18_, neutral alumina, and anhydrous magnesium sulfate. It was then dried with nitrogen and concentrated. The concentrated extracts were separated using an ACQUITY BEH C_18_ column (100 mm×2.1 mm, 1.7 μm) with gradient elution using 0.1% formic acid-5 mmol/L ammonium acetate solution and methanol as mobile phases. The target compounds were detected by ultra-performance liquid chromatography-tandem mass spectrometry with electrospray ionization and positive ion scanning (ESI^+^) in the multiple reaction monitoring (MRM) mode, and quantified by the internal standard method. The linear ranges of *β*_2_-agonists and *β*-blockers were 0.1-20 μg/L, the linear ranges of glucocorticoids were 0.5-200 μg/L, and those of the others were approximately 0.2-50 μg/L. The linear relationships of 50 compounds were good, with correlation coefficients >0.99 in the linear ranges, and limits of quantification (LOQs) in the range of 0.1-0.4 μg/kg. The recoveries of the 50 target compounds spiked in chicken, pork, beef, lamb samples at three levels ranged from 50.3% to 119.9%, while the relative standard deviations (RSDs, *n*=6) ranged from 0.42% to 15.1%. Nine meat samples (including 3 beef, 3 pork, 2 chicken, and duck samples) were tested by this method and the national standard method (GB/T 21981-2008). The *t* test was used for statistical analysis of the hydrocortisone and cortisone contents, and no significant difference was found between the results obtained by the two methods. The developed method was used to analyze 12 beef samples from a farm. In all, 4 compounds were detected, while the other 46 were not detected. The content ranges and detection rates of the compounds were as follows: hydrocortisone: 3.3-22.6 μg/kg, 100%; cortisone: 1.5-2.1 μg/kg, 67%; androstenedione: 0.7-1.2 μg/kg, 17%; and testosterone: 0.6-1.5 μg/kg, 42%. In conclusion, the proposed method is simple, accurate, and sensitive, and hence, is suitable for the detection of foodborne stimulants and progestogens in different kinds of raw meat.

食源性兴奋剂是指来源于食品的兴奋剂,包括一般性食品及保健食品从生产到加工过程中天然存在或故意添加而残留的兴奋剂成分^[1]^。世界反兴奋剂机构(WADA)发布的《2021年禁用清单》^[2]^中明确规定*β*_2_-激动剂、蛋白同化制剂、糖皮质激素、利尿剂等兴奋剂为运动员禁、限用物质。由于食物种类非常多,而且从农田到餐桌供应链条长,食源性兴奋剂的防范难度较大,运动员因食物导致的兴奋剂阳性案例在体育赛事中屡见不鲜^[3,4]^。此外,一些合成类孕激素已经被我国明令禁止,如《食品动物中禁止使用的药品及其他化合物清单(农业农村部公告第250号)》(2019年12月)中规定食用动物中禁止使用醋酸美仑孕酮等激素。为了保障大型体育赛事的成功举办,防止违禁物质污染食物,评估食品中食源性兴奋剂等激素的风险,建立高通量、快速、准确的食源性兴奋剂等激素的检测方法非常必要。

食源性兴奋剂等激素物质的检测方法主要有色谱-质谱法、酶联免疫法、生物芯片等技术^[5,6]^,色谱-质谱法因准确度高、灵敏度高、检测通量高等优势是食源性兴奋剂等激素的主要检测手段,但依然面临较大挑战,主要有以下2点:(1)同类物质极性差异太大,采用同一种检测方法难以获得较好的回收率,比如苯丙酸诺龙的极性较弱,导致样品处理中因为脱脂过程而有较大损失^[7,8]^;(2)对食源性兴奋剂检出限的要求高,通常为痕量甚至是超痕量,且食品基质复杂,基质效应显著^[9]^,样品前处理步骤繁琐。目前传统的固相萃取^[10⇓-12]^和溶剂萃取^[13]^等前处理过程复杂,检测周期较长,近年发展的滤过型净化柱法则存在成本高的问题^[14,15]^。QuEChERS方法已应用在食源性兴奋剂和孕激素的检测中^[16]^,但多数研究仅针对单一种类的物质^[17⇓⇓⇓⇓⇓-23]^,研究肉中兴奋剂高通量多残留检测方法的较少^[24⇓⇓⇓-28]^。本研究以运动员禁、限用兴奋剂和孕激素为研究对象,采用QuEChERS前处理方法,建立了一种同时测定畜禽肉中44种食源性兴奋剂和6种孕激素的超高效液相色谱-串联质谱(UPLC-MS/MS)检测方法,该方法通用性较强,实现了高通量高效率,可为国内外体育赛事中肉类兴奋剂检测提供技术支持。

## 1 实验部分

### 1.1 仪器、材料和试剂

ACQUITY UPLC超高效液相色谱仪、Xevo TQ-S串联四极杆质谱仪,美国WATERS;高速冷冻离心机:GR 22 GIII,日本HITACHI;氮吹仪:N-EVAP^TM^112,配备OA-SYS^TM^水浴加热装置,美国Organomation;多管涡旋混匀仪:MS 200,杭州瑞诚仪器有限公司;涡旋混合器:Vortex-genie 2,美国Scientific Industries;数控超声波清洗器:KQ-500 DE,昆山市超声仪器有限公司。

甲醇、乙腈(色谱纯)均购于美国Fisher Scientific有限公司;无水硫酸镁、氯化钠、中性氧化铝(100~200目)购自国药集团试剂公司;*N*-丙基乙二胺键合固相吸附材料(PSA, 40~60 μm)、十八烷基键合硅胶吸附材料(C_18_, 40~60 μm)、氨基吸附剂(NH_2_, 40~60 μm)均购于博纳艾杰尔有限公司;实验室用水由Milli-Q超纯水净化处理系统制备。

### 1.2 标准溶液的配制

50种被测标准物质(纯度大于95%)和11种内标(100 mg/L的标准溶液)购于美国Sigma-Aldrich、德国Dr. Ehrenstorfer等公司。被测物按照物质的分类用甲醇配制成1~10 mg/L的混合标准储备液,内标用甲醇配制成质量浓度均为100 μg/L的混合内标中间液。置于-20 ℃冷冻保存。

### 1.3 样品处理

#### 1.3.1 提取

准确称取粉碎均质后的肉样品5.0 g于50 mL螺盖离心管中,加入40 μL混合内标中间液,再加入5 mL超纯水,涡旋10 s后,准确加入20 mL含0.5%乙酸的乙腈溶液,振荡提取15 min,加入1.0 g氯化钠和4.0 g无水硫酸镁,快速混匀,继续振荡10 min, 8000 r/min离心5 min。

#### 1.3.2 净化

移取上清液10 mL于另一事先装入净化剂(PSA 165 mg+C_18_ 165 mg+中性氧化铝165 mg+无水硫酸镁900 mg)的螺盖离心管中,振荡混匀10 min, 8000 r/min离心5 min。准确移取上清液5 mL,置于氮吹仪上45 ℃氮气吹干,加入1.0 mL甲醇/水(含0.1%甲酸和5 mmol/L乙酸铵)(1/9, v/v),涡旋复溶,过0.22 μm微孔滤膜后,UPLC-MS/MS待测。

#### 1.3.3 基质空白提取液的制备

取不含被测物的鸡肉样品,不加内标物,按照1.3.1节和1.3.2节的操作处理,得到基质空白提取液,用于配制系列基质标准工作溶液。

### 1.4 仪器条件

#### 1.4.1 液相色谱

色谱柱:Waters Acquity BEH C_18_反相液相色谱柱(100 mm×2.1 mm, 1.7 μm);柱温:30 ℃;进样量:5 μL。流动相:A为甲醇,B为含0.1%甲酸和5 mmol/L乙酸铵的水溶液,流速为0.3 mL/min,梯度洗脱程序:0~0.5 min, 5%A; 0.5~1.0 min, 5%A~10%A; 1.0~6.0 min, 10%A~70%A; 6.0~8.0 min, 70%A~80%A; 8.0~9.0 min, 80%A~100%A; 9.0~10.0 min, 100%A; 10.1~12.0 min, 5%A。

#### 1.4.2 质谱

电喷雾正离子扫描,多反应监测(MRM)模式,毛细管电压:0.5 kV;锥孔电压:30 V;脱溶剂气流速:650 L/h;脱溶剂气温度:450 ℃;锥孔气流速:150 L/h。其他质谱分析参数见表1。

**表1:** 50种目标分析物及11种内标物的保留时间及主要质谱参数

No.	Compound	Retention time/min	Parent ion (*m/z*)	Quantitative/ qualitative ions (*m/z*)	Collision voltages/V	Cone voltage/V
1	tulobuterol (妥布特罗)	4.97	228.4	172.2/154.1	10/16	18
2	clenbuterol (克伦特罗)	4.56	277.4	132.1/203.1	26/14	34
3	bambuterol (班布特罗)	5.12	368.7	72.2/294.4	34/20	18
4	salmeterol (沙美特罗)	7.31	416.7	91.2/380.6	38/18	25
5	clenproperol (克伦丙罗)	2.83	263.3	203.1/168.1	16/26	26
6	zilpaterol (齐帕特罗)	2.82	262.4	185.2/202.2	24/18	18
7	fenoterol (菲诺特罗)	3.37	304.5	107.2/135.2	30/16	4
8	penbuterol (喷布特罗)	7.31	292.5	236.3/74.2	14/20	72
9	clencyclohexerol (克伦塞罗)	3.81	319.4	203.1/81.1	20/26	36
10	mabuterol (马布特罗)	5.02	311.4	217.2/237.2	24/14	20
11	brombuterol (溴布特罗)	4.96	367.4	132.3/293.1	46/18	26
12	cimaterol (西马特罗)	2.64	220.4	160.2/143.2	14/24	18
13	salbutamol (沙丁胺醇)	2.86	240.4	148.2/166.2	16/12	16
14	metaproterenol (奥西那林)	2.20	212.3	152.1/125.2	16/20	22
15	terbutaline (特布他林)	2.80	226.4	152.2/125.1	14/22	30
16	clorprenaline (氯丙那林)	4.51	214.3	154.2/118.9	16/26	18
17	ractopamine (莱克多巴胺)	4.21	302.5	107.2/164.2	26/14	26
18	demethyl coclaurine (去甲乌药碱)	3.32	272.4	107.2/161.2	20/18	40
19	tretoquinol (曲托喹酚)	4.22	346.5	149.1/164.2	50/14	6
20	atenolol (阿替洛尔)	2.87	267.4	145.2/74.2	26/22	20
21	propranolol (普萘洛尔)	5.94	260.4	183.2/116.2	18/16	18
22	metoprolol (美托洛尔)	4.75	268.5	72.1/116.2	18/18	18
23	trenbolone (群勃龙)	6.98	271.4	199.2/165.3	22/44	62
24	methyltestosterone (甲基睾酮)	7.75	303.6	97.1/109.2	26/24	32
25	boldenone (勃地酮)	7.06	287.5	121.1/135.2	26/14	30
26	nortestosterone (诺龙)	7.20	275.1	109.0/257.2	28/16	2
27	stanozolol (司坦唑醇)	8.33	329.6	81.1/95.1	38/38	16
28	methandienone (去氢甲睾酮)	7.31	301.5	121.1/149.2	26/14	26
29	mesterolone (美睾酮)	8.33	305.5	269.4/95.6	16/32	15
30	mestanolone (美雄诺龙)	8.45	305.6	269.4/105.2	14/20	10
31	testosterone (睾酮)	7.49	289.5	97.1/109.2	20/22	48
32	4-androstene-3 (雄烯二酮)	7.19	287.5	97.2/109.2	20/24	34
33	prasterone (脱氢表雄酮)	7.49	289.5	253.3/213.3	10/18	6
34	progesterone (孕酮)	8.40	315.5	97.1/109.2	20/22	12
35	chlormadinone acetate (醋酸氯地孕酮)	8.20	405.1	309.1/345.1	16/14	4
36	melengestrol acetate (醋酸美伦孕酮)	8.30	397.2	279.2/337.1	22/14	2
37	medroxyprogesterone acetate (醋酸甲羟孕酮)	8.25	387.2	123.0/327.2	32/14	2
38	17-*α*-hydroxyprogesterone (17-*α*-羟孕酮)	7.58	331.2	97.0/109.2	26/30	4
39	megestrol (甲地孕酮)	7.87	343.1	187.1/325.1	26/18	2
No.	Compound	Retention time/min	Parent ion (*m/z*)	Quantitative/ qualitative ions (*m/z*)	Collision voltages/V	Cone voltage/V
40	triamterene (氨苯喋啶)	4.43	254.4	141.1/104.2	44/38	16
41	spironolactone (螺内酯)	7.10	341.5	107.2/91.1	26/48	48
42	canrenone (坎利酮)	7.11	341.5	107.2/91.1	28/52	58
43	dexamethasone (地塞米松)	6.78	393.5	373.4/355.3	8/10	12
44	hydrocortisone (氢化可的松)	6.42	363.5	121.1/105.1	22/44	44
45	prednisolone (波尼松龙)	6.42	361.5	325.4/307.4	8/10	24
46	cortisone (可的松)	6.19	361.6	163.2/121.2	24/36	18
47	prednisone (泼尼松)	6.11	359.5	147.1/313.4	26/12	28
48	fluocinolone acetonide (醋酸氟轻松)	6.82	453.5	121.2/433.4	28/8	16
49	fludrocortisone (氟氢可的松)	6.34	381.5	239.3/91.2	24/58	38
50	beclomethasone (倍氯米松)	6.81	409.5	373.4/121.2	8/42	6
I-1	methyltestosterone-D_3_ (甲基睾酮-D_3_)	7.90	306.6	97.2/109.2	24/28	10
I-2	testosterone-D_3_ (睾酮-D_3_)	7.49	292.5	97.2/109.1	24/20	90
I-3	hydrocortisone-D_3_ (氢化可的松-D_3_)	6.42	366.6	121.2/330.4	22/14	16
I-4	salbutamol-D_3_ (沙丁胺醇-D_3_)	2.85	243.5	151.2/169.2	16/12	32
I-5	boldenone--D_3_ (勃地酮-D_3_)	7.05	290.6	121.2/138.1	22/12	12
I-6	clenbuterol-D_9_ (克伦特罗-D_9_)	4.54	286.4	204.1/133.0	16/28	6
I-7	dexamethasone-D_5_ (地塞米松-D_5_)	6.78	398.6	378.5/360.4	8/10	12
I-8	ractopamine-D_5_ (莱克多巴胺-D_5_)	4.19	307.5	165.2/111.1	14/32	14
I-9	progesterone-D_9_ (孕酮-D_9_)	8.35	324.6	100.1/113.2	20/24	26
I-10	demethyl coclaurine-D_4_ (去甲乌药碱-D_4_)	3.28	276.5	108.3/164.3	20/18	18
I-11	tretoquinol-D_9_ (曲托喹酚-D_9_)	4.18	355.6	164.1/137.1	16/46	12

## 2 结果与讨论

### 2.1 UPLC-MS/MS条件选择

#### 2.1.1 质谱条件的优化

分别将100 μg/L单一标准溶液注入离子源,进行一级质谱全扫描,在最佳碰撞电压下确认目标化合物的母离子*m/z*值,然后进行二级质谱优化,通过优化碰撞能量,对碎片离子进行全扫描,选取响应最强与次强的碎片离子作为定量和定性离子,经优化后得到的质谱条件参数见表1。

实验发现所有被测物均在ESI^+^下具有较强的信号值,大部分被测物可获得较高丰度的[M+H]^+^峰。螺内酯的相对分子质量为416.58,但在ESI^+^或ESI^-^模式下都找不到其母离子信息,在*m/z* 341.5处出现了脱去乙酰基巯基的[M-C_2_H_3_OS]^+^峰,该峰信号稳定,可作为螺内酯的一级质谱信号,其二级特征离子信号重现性好,可满足监测要求。

#### 2.1.2 色谱条件的优化

先后采用乙腈、甲醇、水、0.1%甲酸水溶液、含0.1%甲酸和5 mmol/L乙酸铵的水作为流动相,观察标准溶液各个色谱峰的峰形、分离度等。结果发现,甲酸的加入能够明显提高被测物的离子化效率,提高分离度,再加入乙酸铵能够使大部分被测物的峰信号明显增强。用乙腈时较早出峰的*β*_2_-激动剂类在浓度高时峰有分叉现象,甲醇-水(含0.1%甲酸和5 mmol/L乙酸铵)为流动相时所有物质的峰形较好。

试验发现5 cm长的色谱柱无法将同分异构体美睾酮和美雄诺龙的色谱峰分离,最终选择10 cm长的色谱柱。在本实验条件下,被测物中两对具有相同母离子和子离子的同分异构体(美睾酮和美雄诺龙)和同系物(螺内酯和坎利酮)的色谱峰均能够较好的分离,见图1。

**图1:**
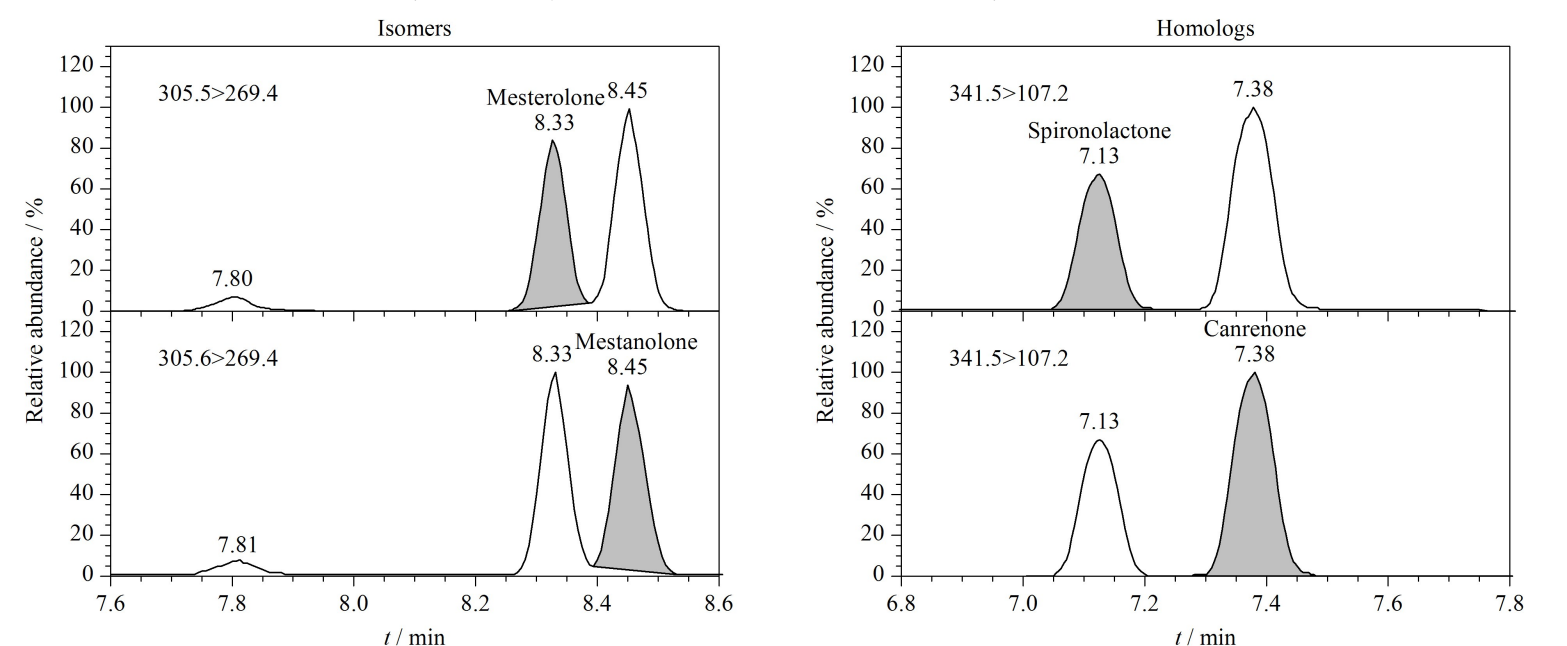
同分异构体(美睾酮和美雄诺龙)和同系物(螺内酯和坎利酮)的色谱图

50种目标化合物及11种内标的MRM色谱图见附图(详见 https://www.chrom-China.com)。

### 2.2 前处理方法的优化

#### 2.2.1 提取溶剂的选择

考察了乙腈中分别加入0、5%、10%、20%、50%的甲醇(均含有0.5%乙酸)6种提取剂的提取效果。随着甲醇在乙腈体系中的比例增大,其蛋白沉淀效果越来越差,在样品前处理-氮吹浓缩过程中可以看到有明显的白色脂类析出,复溶后过滤膜压力大,且溶液混浊,最终选择乙腈作为提取溶剂。

#### 2.2.2 提取溶剂酸度的考察

基于绝大多数目标物均带有酸性基团,采用酸溶液提取更有利于化合物的溶出,分别考察了乙腈中含0.1%、0.5%、1%、2%乙酸的提取效果,结果见图2, *β*_2_-激动剂、*β*-阻断剂类、蛋白同化激素在酸度超过0.5%时,有基质干扰,回收率有降低的趋势;糖皮质激素、孕酮和利尿剂对提取液中乙酸的比例不敏感。当采用含0.5%乙酸的乙腈溶液作为取溶剂时,所有被测物的回收率均大于50%,确定为最优提取剂。

**图2:**
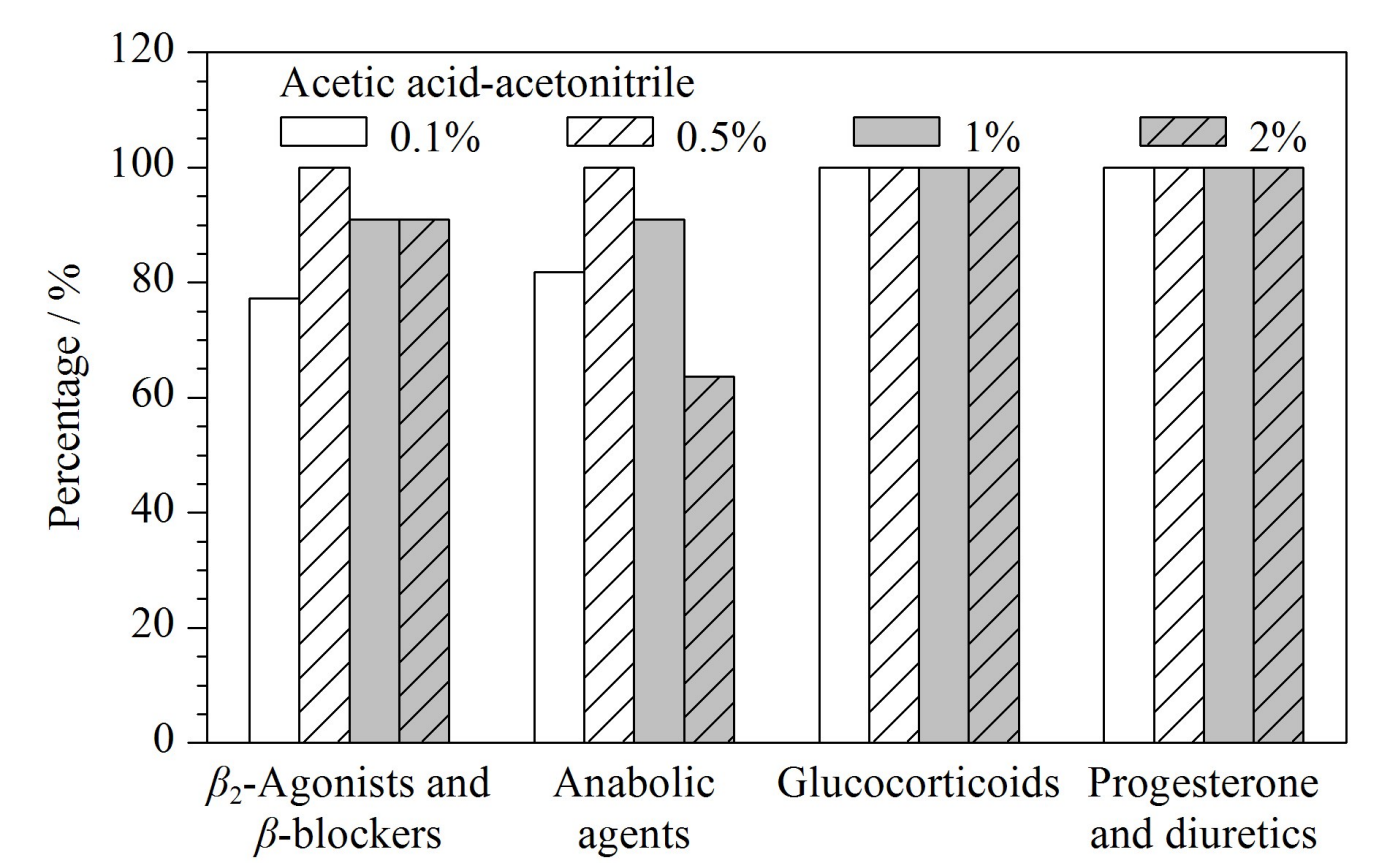
采用含不同量乙酸的乙腈做提取液时回收率大于 50%的化合物个数占该类化合物总个数的百分比(基质为鸡肉)

#### 2.2.3 样品净化剂的选择

对QuEChERS方法中常用的4种吸附剂(PSA、C_18_、NH_2_、中性氧化铝)单独及组合使用的净化效果和吸附作用进行了考察。

单独采用NH_2_吸附剂时,大约有45%的化合物回收率比使用其他3种净化剂时低,且基线噪声较大,干扰峰多,表明NH_2_对被测物有吸附作用,净化效果不理想;PSA、C_18_和中性氧化铝对目标化合物的吸附相对较小,提取回收良好,且无明显杂质峰干扰。

进一步采用以下4种组合方案:① PSA 250 mg+C_18_ 250 mg; ② PSA 250 mg+中性氧化铝250 mg; ③ C_18_ 250 mg+中性氧化铝250 mg; ④ PSA 165 mg+C_18_ 165 mg+中性氧化铝165 mg,考察鸡肉基质中的加标回收率,见图3。结果表明,采用净化剂组合①时*β*-兴奋剂类回收率最高,但蛋白同化激素、糖皮质激素和孕激素的回收率低;采用组合②或③时,蛋白同化激素和糖皮质激素回收率较高,但对*β*-兴奋剂类和孕激素回收率不理想;组合④ PSA+C_18_+Al_2_O_3_ 3种复配时,糖皮质激素的回收率较高,且被测物回收率低于20%的最少。

**图3:**
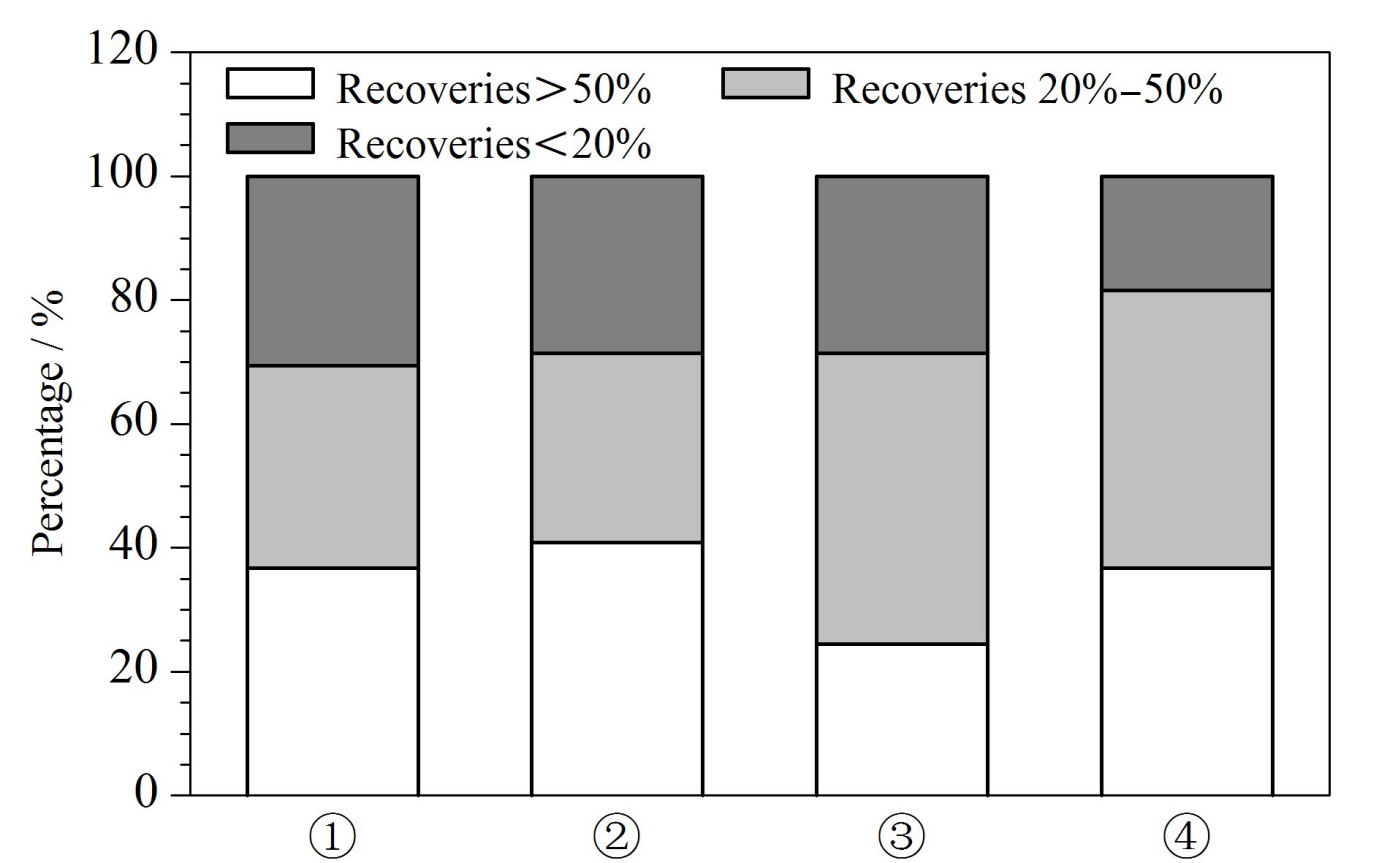
采用不同净化剂时鸡肉基质中不同回收率范围的 目标物个数占总被测物个数的比例

综合以上多重因素,本试验最终采用组合④作为最终净化剂。其中PSA可以去除肉中的脂肪酸类和糖类干扰,C_18_则对脂肪和非极性化合物的吸附效果比较明显,中性氧化铝容易吸附杂环类、芳香烃和有机胺等富电化合物,3种联合使用达到净化的目的,最终上机溶液为澄清透明溶液。

#### 2.2.4 复溶溶剂的选择

根据流动相和各目标化合物的溶解性能,考察了3种复溶溶剂:①乙腈/水(含0.1%甲酸)(1/9, v/v); ②甲醇/水(含0.1%甲酸)(1/9, v/v); ③甲醇/水(含0.1%甲酸和5 mmol/L乙酸铵)(1/9, v/v)。通过比较发现,当采用溶剂①稀释标准溶液或样品时,会出现明显峰分叉现象,而采用②时则峰形很好,由于使用的流动相中有0.1%甲酸和5 mmol/L乙酸铵水溶液,又考察了溶剂③,结果发现溶剂②和③没有明显差别,最终采用溶剂③作为复溶溶剂。

#### 2.2.5 对应内标物的选择

对内标化合物与被测物的匹配进行了选择,以被测物和内标物结构类似、色谱峰保留时间相近、回收率接近为原则,选择最为合适的内标物。最终确定11种内标物对应50种被测物,具体见表2。

**表2:** 50种被测物对应的内标化合物、线性范围、线性方程、相关系数、检出限、定量限及基质效应(SSE)

No.	Compound	Internal standard^*^	Linear range/ (μg/L)	Linear equation	*R*^2^	LOD/ (μg/kg)	LOQ/ (μg/kg)	SSE/ %
1	tulobuterol	clenbuterol-D_9_	0.1-20	*y*=1.008*x*+0.0127	0.9992	0.03	0.1	109
2	clenbuterol	clenbuterol-D_9_	0.1-20	*y*=1.259*x*+0.0982	0.9994	0.03	0.1	97
3	bambuterol	clenbuterol-D_9_	0.1-20	*y*=0.415*x*-0.0548	0.9995	0.03	0.1	148
4	salmeterol	ractopamine-D_5_	0.1-20	*y*=0.339*x*-0.0420	0.9980	0.03	0.1	133
5	clenproperol	clenbuterol-D_9_	0.2-20	*y*=0.725*x*+0.0577	0.9992	0.07	0.2	108
6	zilpaterol	salbutamol-D_3_	0.1-20	*y*=0.921*x*+0.163	0.9975	0.03	0.1	85
7	fenoterol	salbutamol-D_3_	0.1-20	*y*=1.119*x*-0.0307	0.9992	0.03	0.1	104
8	penbuterol	clenbuterol-D_9_	0.1-10	*y*=4.360*x*+0.259	0.9961	0.03	0.1	129
9	clencyclohexerol	clenbuterol-D_9_	0.1-20	*y*=1.324*x*+0.132	0.9985	0.03	0.1	80
10	mabuterol	clenbuterol-D_9_	0.2-20	*y*=0.116*x*+0.00952	0.9991	0.07	0.2	108
11	brombuterol	clenbuterol-D_9_	0.1-20	*y*=0.137*x*-0.00338	0.9993	0.03	0.1	124
12	cimaterol	clenbuterol-D_9_	0.2-20	*y*=1.042*x*+0.0599	0.9996	0.07	0.2	117
13	salbutamol	salbutamol-D_3_	0.1-10	*y*=0.432*x*+0.0494	0.9971	0.03	0.1	105
14	metaproterenol	clenbuterol-D_9_	0.1-20	*y*=2.046*x*-0.0725	0.9993	0.03	0.1	103
15	terbutaline	clenbuterol-D_9_	0.1-10	*y*=3.548*x*+0.159	0.9987	0.03	0.1	138
16	clorprenaline	clenbuterol-D_9_	0.1-20	*y*=0.752*x*+0.0253	0.9997	0.03	0.1	177
17	ractopamine	ractopamine-D_5_	0.1-20	*y*=1.121*x*+0.390	0.9989	0.03	0.1	92
18	demethyl coclaurine	demethyl coclaurine-D_4_	0.1-10	*y*=3.200*x*+0.0441	0.9999	0.03	0.1	92
19	tretoquinol	tretoquinol-D_9_	0.1-20	*y*=0.146*x*-0.00404	0.9994	0.03	0.1	115
20	atenolol	clenbuterol-D_9_	0.1-20	*y*=1.789*x*+0.333	0.9959	0.03	0.1	127
21	propranolol	clenbuterol-D_9_	0.1-20	*y*=1.761*x*+0.0714	0.9996	0.03	0.1	87
22	metoprolol	clenbuterol-D_9_	0.1-20	*y*=1.137*x*+0.0171	0.9999	0.03	0.1	75
23	trenbolone	boldenone-D_3_	0.2-50	*y*=2.423*x*+1.230	0.9951	0.07	0.2	112
24	methyltestosterone	methyltestosterone-D_3_	0.2-50	*y*=0.737*x*+0.175	0.9957	0.07	0.2	90
25	boldenone	boldenone-D_3_	0.2-50	*y*=3.571*x*+0.923	0.9971	0.07	0.2	85
26	nortestosterone	testosterone-D_3_	0.2-50	*y*=0.0158*x*+0.0105	0.9963	0.07	0.2	106
27	stanozolol	progesterone-D_9_	0.2-50	*y*=1.858*x*-0.138	0.9987	0.07	0.2	103
28	methandienone	testosterone-D_3_	0.2-50	*y*=0.022*x*+0.0111	0.9951	0.07	0.2	95
29	mesterolone	methyltestosterone-D_3_	0.2-50	*y*=0.0284*x*+0.0152	0.9966	0.07	0.2	173
30	mestanolone	methyltestosterone-D_3_	0.2-50	*y*=0.0274*x*-0.0303	0.9957	0.07	0.2	95
31	testosterone	testosterone-D_3_	0.2-50	*y*=0.0428*x*+0.0098	0.9966	0.07	0.2	98
32	4-androstene-3	boldenone-D_3_	0.2-50	*y*=0.642*x*+0.0248	0.9995	0.07	0.2	99
33	prasterone	boldenone-D_3_	0.2-50	*y*=0.135*x*+0.175	0.9982	0.07	0.2	123
34	progesterone	progesterone-D_9_	0.2-50	*y*=0.556*x*+0.0887	0.9983	0.07	0.2	89
35	chlormadinone acetate	progesterone-D_9_	0.2-50	*y*=0.00469*x*-0.0017	0.9987	0.07	0.2	127
36	melengestrol acetate	progesterone-D_9_	0.2-50	*y*=0.0538*x*+0.0016	0.9998	0.07	0.2	123
37	medroxyprogesterone acetate	progesterone-D_9_	0.2-50	*y*=0.0671*x*-0.0176	0.9987	0.07	0.2	123
38	17-*α*-hydroxyprogesterone	testosterone-D_3_	0.2-50	*y*=0.158*x*-0.0587	0.9940	0.07	0.2	107
39	megestrol	methyltestosterone-D_3_	0.2-50	*y*=0.0298*x*+0.0062	0.9976	0.07	0.2	118
40	triamterene	ractopamine-D_5_	0.2-50	*y*=0.998*x*+1.474	0.9993	0.07	0.2	108
41	spironolactone	boldenone-D_3_	0.2-50	*y*=0.980*x*+0.112	0.9992	0.07	0.2	104
42	canrenone	testosterone-D_3_	0.2-50	*y*=0.0116*x*+0.0023	0.9972	0.07	0.2	99
43	dexamethasone	dexamethasone-D_5_	0.5-200	*y*=2.269*x*+0.170	0.9995	0.12	0.4	94
44	hydrocortisone	hydrocortisone-D_3_	0.5-100	*y*=0.630*x*+0.044	0.9991	0.12	0.4	99
45	prednisolone	hydrocortisone-D_3_	0.5-200	*y*=0.131*x*+0.0.038	0.9964	0.12	0.4	85
46	cortisone	hydrocortisone-D_3_	0.5-100	*y*=0.894*x*+0.056	0.9986	0.12	0.4	82
47	prednisone	hydrocortisone-D_3_	0.5-100	*y*=0.272*x*-0.00203	0.9996	0.12	0.4	91
48	fluocinolone acetonide	hydrocortisone-D_3_	0.5-100	*y*=0.446*x*+0.0587	0.9934	0.12	0.4	80
49	fludrocortisone	hydrocortisone-D_3_	0.5-100	*y*=0.218*x*-0.00661	0.9999	0.12	0.4	81
50	beclomethasone	hydrocortisone-D_3_	0.5-100	*y*=0.0535*x*+0.0115	0.9992	0.12	0.4	60

* Mass concentration of each internal standard is 1 μg/L. *y*: ratio of the peak area of the quantitative ion pair of the compound to the peak area of the internal standard quantitative ion pair. *x*: mass concentration of the compound, μg/L.

#### 2.2.6 基质效应的考察

为了评价基质效应,本研究采用基质标准曲线斜率与溶剂标准曲线斜率的比值(signal suppression/enhancement, SSE)考察了目标物在基质中的信号增强或抑制的程度。SSE值在80%~120%之间表明基质效应影响不大,当其高于120%时显示基质增强效应,当其低于80%时显示基质抑制效应。本试验以鸡肉为基质考察了其SSE值,见表2。有2种化合物有基质抑制效应,有12种化合物有基质增强效应,其余36种化合物无显著影响。为了消除基质的影响,采用基质空白提取液稀释标准溶液配制标准曲线。

### 2.3 方法的线性关系与灵敏度

采用基质空白提取液稀释标准溶液配制基质标准溶液,以被测化合物定量离子对的峰面积与内标定量离子对峰面积比为纵坐标(*y*),以被测物的质量浓度为横坐标(*x*, μg/L)绘制标准工作曲线,考察被测物的线性关系,结果见表2, *β*_2_-激动剂类(No. 1~19)和*β*-阻断剂类(No. 20~22)的线性范围是0.1~20 μg/L,蛋白同化激素类(No. 23~33)、孕激素类(No. 34~39)、利尿剂类(No. 40~42)的线性范围是0.2~50 μg/L,糖皮质激素类(No. 43~50)的线性范围0.5~200 μg/L。所有目标分析物在其线性范围内线性拟合良好,线性相关系数(*R*^2^)均超过0.99。

由于定性离子对比定量离子对的信号弱,为了确保能够准确定性,本试验以定性离子对的3倍信噪比(*S/N*=3)对应的浓度作为检出限,*S/N*=10对应的浓度作为定量限,见表2。本方法的检出限范围为0.03~0.12 μg/kg,定量限范围为0.1~0.4 μg/kg。庄玥等^[25]^建立的方法与本研究所建方法和被测物类似,是将待测样品先经乙酸乙酯提取,再经氮气吹干乙腈定容,QuEChERS(45 mg PSA+25 mg C_18_+50 mg无水硫酸镁)净化后以HPLC-MS/MS检测,该方法定量限为0.1~1 μg/kg。国家标准方法GB/T 21981-2008《动物源食品中激素多残留检测方法 液相色谱-质谱/质谱法》的定量限为0.4~2.0 μg/kg。与上述两种方法相比本方法的定量限更低。

### 2.4 方法准确度和精确度

分别考察了鸡肉、猪肉、羊肉、牛肉基质中目标化合物的加标回收率和精密度(RSD),并同时测定相应的基质空白样品。结果见表3~6,鸡肉的加标回收率范围50.3%~114.7%,精密度范围0.42%~15.1%;猪肉的加标回收率范围50.3%~116.3%,精密度范围0.58%~13.1%;羊肉的加标回收率范围52.0%~119.9%,精密度范围0.50%~15.0%;牛肉的加标回收率范围50.4%~117.7%,精密度范围0.76%~13.2%。尽管内标在整个试验过程中对目标分析物的定量都起着纠偏和校正的作用,但是仍有3种化合物司坦唑醇、美睾酮、美雄诺龙的回收率较低(50%~60%),其他化合物的回收率良好(>60%)。

**表3:** 鸡肉中目标物的加标回收率及精密度(*n*=6)

No.	Compound	Matrix content/ (μg/kg)	Added levels^*^										
			Low			Middle				High			
			Recovery/%	RSD/%		Recovery/%		RSD/%			Recovery/%	RSD/%
1	tulobuterol	-	73.0	8.55		93.0		7.05		98.7		5.10
2	clenbuterol	-	70.0	14.3		92.0		6.61		114.7		10.7
3	bambuterol	-	77.0	7.90		93.3		9.66		106.7		1.08
4	salmeterol	-	90.0	11.1		99.0		3.50		92.7		4.49
5	clenproperol	-	-	-		74.3		3.88		85.3		1.35
6	zilpaterol	-	66.7	8.66		78.3		8.97		102.0		8.55
7	fenoterol	-	72.7	4.20		78.0		2.56		92.2		1.04
8	penbuterol	-	65.0	7.69		80.3		10.1		93.3		9.90
9	clencyclohexerol	-	62.3	4.04		70.7		10.6		101.3		10.9
10	mabuterol	-	-	-		96.7		8.68		102.0		1.96
11	brombuterol	-	83.0	13.6		94.7		8.07		92.7		1.25
12	cimaterol	-	-	-		114.3		1.82		106.0		3.27
13	salbutamol	-	76.7	7.53		95.0		7.37		91.0		1.10
14	metaproterenol	-	62.0	5.59		65.0		4.62		76.0		0.76
15	terbutaline	-	80.0	12.5		100.7		8.45		101.7		2.48
16	clorprenaline	-	80.3	13.1		92.0		9.29		102.7		2.98
17	ractopamine	-	83.3	6.93		94.0		7.45		108.7		3.83
18	demethyl coclaurine	-	98.7	5.21		103.2		3.02		112.3		5.05
19	tretoquinol	-	95.2	6.02		103.5		5.33		110.5		2.04
20	atenolol	-	83.7	7.59		87.3		8.28		107.3		3.88
21	propranolol	-	86.7	13.3		86.3		13.4		106.7		12.1
22	metoprolol	-	76.7	15.1		92.0		9.78		108.7		5.92
23	trenbolone	-	72.7	3.18		72.1		0.58		76.8		2.13
24	methyltestosterone	-	73.3	1.57		108.1		5.98		94.6		2.65
25	boldenone	-	79.3	5.25		100.6		7.75		96.0		2.60
26	nortestosterone	-	112.0	1.79		113.3		4.08		105.8		3.15
27	stanozolol		104.3	2.77		87.2		0.92		73.8		3.45
28	methandienone	-	104.7	3.98		103.5		2.30		105.9		3.21
29	mesterolone	-	52.0	3.85		52.3		9.23		50.4		8.40
30	mestanolone	-	52.3	7.72		55.4		10.2		50.3		10.2
31	testosterone	-	84.7	3.61		89.3		7.71		97.9		6.14
32	4-androstene-3	-	103.0	2.57		103.1		1.81		93.0		2.78
33	prasterone	-	74.7	0.77		64.0		6.12		74.5		4.37
34	progesterone		104.7	3.86		113.1		10.5		92.0		0.87
35	chlormadinone acetate	-	84.0	2.38		82.4		5.55		86.1		0.76
36	melengestrol acetate	-	93.3	8.66		85.1		9.87		93.5		7.92
37	medroxyprogesterone acetate	-	96.0	2.08		87.4		1.05		88.5		0.94
38	17-*α*-hydroxyprogesterone	-	103.3	5.59		93.3		6.19		101.3		6.57
39	megestrol	-	88.3	8.65		63.3		9.12		73.7		7.84
40	triamterene	-	62.7	4.02		74.4		9.17		66.0		9.09
41	spironolactone	-	62.3	2.45		67.9		7.06		67.3		3.74
42	canrenone	-	90.0	11.1		100.0		7.00		72.0		5.56
43	dexamethasone	-	95.7	5.76		106.7		0.89		103.5		2.17
44	hydrocortisone	-	112.7	3.36		88.7		3.17		97.2		1.52
45	prednisolone	-	85.3	11.1		93.6		10.1		83.8		3.58
46	cortisone	-	78.3	7.03		71.9		0.42		73.3		5.51
47	prednisone	-	90.0	5.09		72.7		2.21		76.5		7.98
48	fluocinolone acetonide	-	85.3	1.35		85.7		5.39		66.3		2.51
49	fludrocortisone	-	91.7	4.92		80.8		5.74		62.4		4.49
50	beclomethasone	-	70.3	9.16		68.2		8.69		72.5		6.69

-: not detected. * Standard addition levels were 0.1, 0.8, 2 μg/kg for compounds No. 1-22; 0.5, 4, 10 μg/kg for the compounds No. 23-42; 1, 8, 20 μg/kg for the compounds No. 43-50.

**表4:** 猪肉中目标物的加标回收率及精密度(*n*=6)

No.	Compound	Matrix content/ (μg/kg)	Added levels^*^										
			Low			Middle				High			
			Recovery/%	RSD/%		Recovery/%		RSD/%			Recovery/%	RSD/%
1	tulobuterol	-	93.3	6.19		100.3		0.58		105.7		3.82
2	clenbuterol	-	92.0	7.84		105.0		4.36		110.7		5.52
3	bambuterol	-	92.0	3.77		92.0		6.79		104.0		1.92
4	salmeterol	-	92.7	5.10		90.7		3.37		95.3		1.21
5	clenproperol	-	-	-		116.3		2.76		107.3		3.53
6	zilpaterol	-	84.7	3.61		100.0		10.0		103.3		4.03
7	fenoterol	-	97.8	1.97		72.2		9.61		84.4		12.8
8	penbuterol	-	85.3	2.95		87.7		7.76		110.0		6.56
9	clencyclohexerol	-	93.7	5.05		92.7		4.98		108.7		6.46
10	mabuterol	-	-	-		97.7		11.0		85.0		5.13
11	brombuterol	-	87.0	6.99		81.3		1.88		86.0		2.33
12	cimaterol	-	-	-		107.3		4.30		112.7		4.19
13	salbutamol	-	93.3	4.83		110.7		8.15		114.0		1.75
14	metaproterenol	-	107.0	4.07		99.7		10.0		112.0		3.57
15	terbutaline	-	110.3	2.09		91.0		8.30		86.7		2.90
16	clorprenaline	-	100.0	1.34		110.0		1.76		111.3		4.52
17	ractopamine	-	75.3	5.03		66.7		8.66		62.7		9.75
18	demethyl coclaurine	-	95.3	4.73		105.7		2.73		110.7		3.09
19	tretoquinol	-	84.0	4.76		89.3		7.28		93.7		5.88
20	atenolol	-	96.0	5.51		110.0		1.69		110.7		2.09
21	propranolol	-	98.8	6.21		97.5		5.28		106.3		3.32
22	metoprolol	-	95.1	4.02		97.6		5.33		113.2		5.71
21	trenbolone	-	76.7	7.53		77.3		1.49		77.6		3.57
24	methyltestosterone	-	97.8	1.97		98.7		7.67		105.3		2.32
25	boldenone	-	113.3	10.2		102.7		2.98		104.0		4.62
26	nortestosterone	-	114.0	3.04		113.4		3.12		115.8		2.88
27	stanozolol	-	65.3	7.70		66.7		4.58		70.8		10.6
28	methandienone	-	103.3	5.59		100.7		4.14		105.9		3.21
29	mesterolone	-	51.0	7.07		51.3		5.95		50.4		8.40
30	mestanolone	-	50.3	3.03		53.7		9.19		57.7		4.36
31	testosterone	-	75.0	6.67		103.3		1.12		97.9		6.14
32	4-androstene-3	-	113.3	5.09		113.0		3.86		99.1		4.45
33	prasterone	-	61.3	3.77		64.0		4.13		66.3		4.61
34	progesterone	-	93.3	12.4		98.7		6.59		92.0		0.87
35	chlormadinone acetate	-	70.0	4.01		69.7		3.61		66.1		0.99
36	melengestrol acetate	-	96.7	5.97		84.0		12.6		83.5		10.9
37	medroxyprogesterone acetate	-	68.3	11.2		72.7		4.20		78.5		1.06
38	17-*α*-hydroxyprogesterone	-	79.0	8.86		69.4		9.32		74.6		7.34
39	megestrol	-	70.3	7.83		69.3		7.08		87.4		5.15
40	triamterene	-	65.0	10.8		65.0		7.69		66.0		9.09
41	spironolactone	-	69.3	1.67		72.0		8.45		74.0		7.15
42	canrenone	-	100.0	5.04		73.3		7.87		72.0		5.56
43	dexamethasone	-	116.7	4.95		105.7		3.04		78.9		4.79
44	hydrocortisone	13.3	87.3	7.36		105.7		5.21		70.8		9.09
45	prednisolone	-	60.0	5.96		73.3		9.86		63.6		4.75
46	cortisone	3.86	62.0	8.06		96.4		7.86		73.1		5.92
47	prednisone	-	66.0	8.44		74.3		7.41		76.5		7.98
48	fluocinolone acetonide	-	65.3	6.37		82.0		6.34		66.3		2.51
49	fludrocortisone	-	87.3	7.36		87.3		11.6		62.4		4.49
50	beclomethasone	-	77.6	10.3		81.1		1.05		69.1		6.41

-: not detected. * Standard addition levels were 0.1, 0.8, 2 μg/kg for compounds No. 1-22; 0.5, 4, 10 μg/kg for the compounds No. 23-42; 1, 8, 20 μg/kg for the compounds No. 43-50.

**表5:** 羊肉中目标物的加标回收率及精密度(*n*=6)

No.	Compound	Matrix content/ (μg/kg)	Added levels^*^										
			Low			Middle				High			
			Recovery/%	RSD/%		Recovery/%		RSD/%			Recovery/%	RSD/%
1	tulobuterol	-	111.0	0.90		104.3		2.21		102.8		1.08
2	clenbuterol	-	116.0	3.95		95.0		3.80		107.8		9.55
3	bambuterol	-	105.0	5.79		113.0		4.06		104.7		6.25
4	salmeterol	-	102.7	14.6		70.3		0.82		80.1		2.31
5	clenproperol	-	-	-		66.0		3.03		72.7		5.39
6	zilpaterol	-	110.0	4.72		65.3		9.96		74.0		14.2
7	fenoterol	-	65.6	10.6		66.7		4.58		76.1		4.99
8	penbuterol	-	114.3	0.50		76.7		2.72		70.0		13.1
9	clencyclohexerol	-	104.0	10.7		67.3		10.1		77.3		13.6
10	mabuterol	-	-	-		110.0		7.10		111.8		9.30
11	brombuterol	-	115.7	2.78		99.0		12.7		103.5		8.40
12	cimaterol	-	-	-		98.0		12.8		96.7		10.0
13	salbutamol	-	110.7	4.46		77.3		13.2		68.9		10.1
14	metaproterenol	-	100.7	4.90		85.7		7.13		87.5		8.66
15	terbutaline	-	96.0	1.04		104.3		11.4		112.7		6.96
16	clorprenaline	-	101.0	0.99		116.0		2.28		113.7		2.38
17	ractopamine	-	74.3	2.05		77.3		2.69		82.3		3.00
18	demethyl coclaurine	-	84.0	1.19		85.3		2.95		79.3		4.68
19	tretoquinol	-	94.0	1.06		102.7		2.98		104.5		2.15
20	atenolol	-	82.7	2.52		90.0		2.22		98.9		11.1
21	propranolol	-	96.0	5.38		101.4		5.08		105.6		4.03
22	metoprolol	-	102.5	4.99		107.3		5.07		112.5		3.22
23	trenbolone	-	69.3	4.41		79.7		10.5		84.3		11.3
24	methyltestosterone	-	108.7	3.83		101.6		2.86		107.9		13.3
25	boldenone	-	98.0	5.40		108.8		4.69		108.1		2.57
26	nortestosterone	-	113.3	1.02		107.8		2.45		107.1		1.77
27	stanozolol	-	70.7	9.94		55.1		4.67		55.9		8.23
28	methandienone	-	84.7	5.94		106.7		3.56		106.4		7.73
29	mesterolone	-	54.0	6.42		54.4		6.07		55.1		5.29
30	mestanolone	-	60.3	11.0		54.3		6.08		52.0		5.75
31	testosterone	1.00	116.7	3.96		100.4		2.74		86.3		12.7
32	4-androstene-3	-	114.7	5.81		91.3		7.29		94.9		3.58
33	prasterone	-	89.0	9.20		64.2		13.9		80.6		12.6
34	progesterone	-	96.3	7.79		80.3		3.61		81.0		13.8
35	chlormadinone acetate	-	67.5	10.1		80.1		7.22		81.6		2.62
36	melengestrol acetate	-	78.8	2.01		78.5		1.93		80.4		9.68
37	medroxyprogesterone acetate	-	64.5	5.74		119.2		3.35		113.8		2.14
38	17-*α*-hydroxyprogesterone		74.6	7.34		119.9		4.83		115.3		2.92
39	megestrol		87.4	5.15		66.8		10.6		66.5		10.8
40	triamterene	-	67.3	6.18		61.2		5.89		72.1		12.0
41	spironolactone	-	79.9	5.95		78.5		8.60		78.7		8.30
42	canrenone	-	71.0	2.82		105.7		2.38		105.5		11.2
43	dexamethasone	-	99.0	15.0		105.9		7.60		104.8		2.75
44	hydrocortisone	4.90	118.3	4.34		90.8		4.98		71.4		0.87
45	prednisolone	-	62.3	7.58		78.5		1.75		75.5		5.53
46	cortisone	1.10	89.0	4.05		80.5		6.29		69.7		5.22
47	prednisone	-	86.7	6.56		72.3		9.10		77.3		3.42
48	fluocinolone acetonide	-	63.0	11.4		84.9		2.35		69.3		10.7
49	fludrocortisone	-	65.7	0.88		86.0		1.71		63.2		7.14
50	beclomethasone	-	74.2	6.77		82.4		4.98		69.0		4.07

-: not detected. * Standard addition levels were 0.1, 0.8, 2 μg/kg for compounds No. 1-22; 0.5, 4, 10 μg/kg for the compounds No. 23-42; 1, 8, 20 μg/kg for the compounds No. 43-50.

**表6:** 牛肉中目标物的加标回收率及精密度(*n*=6)

No.	Compound	Matrix content/ (μg/kg)	Added levels^*^										
			Low			Middle				High			
			Recovery/%	RSD/%		Recovery/%		RSD/%			Recovery/%	RSD/%
1	tulobuterol	-	102.3	2.26		105.3		3.09		89.9		5.33
2	clenbuterol	-	113.7	2.83		111.5		7.83		113.9		5.31
3	bambuterol	-	106.0	2.50		104.7		3.79		89.3		4.77
4	salmeterol	-	87.7	2.37		98.6		3.03		97.3		13.2
5	clenproperol	-	85.7	1.35		85.1		1.49		86.8		4.38
6	zilpaterol	-	101.3	1.51		97.9		1.59		89.6		0.76
7	fenoterol	-	72.0	2.78		80.9		4.59		73.9		5.34
8	penbuterol	-	104.0	3.47		113.5		2.91		109.5		8.45
9	clencyclohexerol	-	65.0	2.66		66.6		8.46		66.6		4.78
10	mabuterol	-	107.7	3.75		117.7		1.44		110.3		0.82
11	brombuterol	-	103.0	0.97		104.8		2.98		107.1		2.68
12	cimaterol	-	112.3	4.39		103.9		0.99		105.6		2.18
13	salbutamol	-	94.3	11.7		106.1		8.12		109.0		2.70
14	metaproterenol	-	67.0	8.31		84.2		4.91		84.2		2.38
15	terbutaline	-	95.0	5.26		102.3		8.64		97.5		10.3
16	clorprenaline	-	85.0	4.71		87.9		1.25		85.0		1.19
17	ractopamine	-	101.0	0.99		86.2		4.96		87.1		2.52
18	demethyl coclaurine	-	93.7	2.47		88.9		3.64		89.7		2.77
19	tretoquinol	-	84.3	3.81		84.1		1.80		87.0		1.59
20	atenolol	-	101.0	3.43		110.2		6.72		104.5		3.78
21	propranolol	-	92.6	4.52		99.8		7.01		100.6		5.01
22	metoprolol	-	96.6	8.75		110.5		4.22		112.9		4.47
23	trenbolone	-	92.3	5.20		102.7		2.97		91.9		1.78
24	methyltestosterone	-	107.5	6.51		111.8		4.19		74.8		2.79
25	boldenone	-	105.3	5.16		116.5		4.41		96.0		2.10
26	nortestosterone	-	86.9	8.31		90.9		4.19		78.8		2.64
27	stanozolol	-	51.7	5.58		54.4		2.57		52.5		3.25
28	methandienone	-	84.3	4.87		99.1		3.07		69.0		3.72
29	mesterolone	-	57.3	4.01		50.4		5.34		53.9		3.18
30	mestanolone	-	53.1	5.50		52.4		5.86		53.9		3.36
31	testosterone	-	70.1	5.44		65.8		7.24		66.0		1.60
32	4-androstene-3	-	92.3	8.40		80.6		7.89		79.1		0.77
33	prasterone	-	92.2	5.92		101.2		7.18		107.5		2.18
34	progesterone	12.0	104.6	1.99		96.6		1.47		93.0		3.22
35	chlormadinone acetate	-	63.7	7.66		75.3		5.81		68.3		9.47
36	melengestrol acetate	-	85.3	12.8		83.4		12.6		83.7		8.31
37	medroxyprogesterone acetate	-	90.0	7.95		93.9		3.31		97.4		3.41
38	17-*α*-hydroxyprogesterone	-	73.4	8.58		76.1		10.1		72.2		5.32
39	megestrol	-	60.7	8.30		71.0		4.09		74.7		3.09
40	triamterene	-	63.7	6.27		61.1		1.36		68.0		2.15
41	spironolactone	-	91.4	7.03		83.7		1.97		86.2		5.03
42	canrenone	-	67.3	5.22		69.1		3.95		66.5		2.90
43	dexamethasone	-	81.2	7.10		107.8		3.03		101.2		10.1
44	hydrocortisone	5.57	88.0	2.11		94.1		6.97		70.4		4.53
45	prednisolone	-	70.3	11.9		71.7		7.36		66.2		8.19
46	cortisone	-	98.6	7.98		90.7		4.83		84.0		3.15
47	prednisone	-	75.4	7.86		79.9		7.70		67.4		7.49
48	fluocinolone acetonide	-	73.0	10.2		78.7		8.51		66.0		9.31
49	fludrocortisone	-	77.0	3.66		78.2		6.70		66.5		7.52
50	beclomethasone	-	66.1	5.21		66.7		6.19		69.1		6.11

-: not detected. * Standard addition levels were 0.8, 4, 8 μg/kg for compounds No. 1-22; 4, 20, 40 μg/kg for the compounds No. 23-42; 8, 40, 80 μg/kg for the compounds No. 43-50.

结果还发现,畜禽肉中常含有一些内源性激素,例如本实验所采用的4种基质,除鸡肉外,其他3种基质中均检出了激素:氢化可的松(猪肉、羊肉、牛肉)、可的松(猪肉、羊肉)、睾酮(羊肉)、孕酮(牛肉),因此推荐用鸡肉做基质空白配制标准溶液。

### 2.5 方法比对及样品的测定

#### 2.5.1 方法比对

同时采用本方法和国家标准方法GB/T 21981-2008测定了9个市售肉类样品。验证的被测物(两个方法重合的被测物)有21个:群勃龙、勃地酮、诺龙、雄烯二酮、美雄酮、睾酮、脱氢表雄酮(普拉雄酮)、甲睾酮、康力龙、美睾酮、美雄诺龙、17-α-羟孕酮、醋酸氯地孕酮、孕酮、醋酸甲羟孕酮、波尼松、可的松、氢化可的松、波尼松龙、地塞米松、倍氯米松。测定结果见表7,除氢化可的松、可的松、雄烯二酮外其他物质均未检出。

**表7:** 肉类样品采用两种方法检测的结果(*n*=2)

Sample	This method/(μg/kg)				GB/T 21981-2008/(μg/kg)			
	Hydrocortisone	Cortisone	4-Androstene-3		Hydrocortisone		Cortisone	4-Androstene-3
Pork 1	4.9	1.7	-		5.2		1.7	-
Pork 2	16.4	7.7	-		18.4		8.1	-
Pork 3	5.6	0.9	-		6.7		1.4	-
Chicken breast	-	-	-		-		-	-
Chicken wings	-	-	-		-		-	-
Duck	-	-	-		-		-	-
Beef 1	5.9	0.4	-		6.3		0.4	-
Beef 2	3.8	1.3	0.3		3.4		1.1	-
Beef 3	5.8	2.4	-		5.7		2.0	-
LOQ/(μg/kg)	0.4	0.4	0.2		0.4		0.4	0.4

-: not detected.

为了分析比对自建方法和国标方法的一致性,针对氢化可的松和可的松的12组检测数据(见表7),采用*t*检验进行统计学分析,计算本方法所得结果与国标方法所得结果的差值,选择*α*=5%显著性水平作为判断标准,当*n*=6时,查*t*分布双侧临界值表*t*_(0.05,_*_n_*_-1)_=2.571,经计算得到*t*_氢化可的松_=1.62, *t*_可的松_=0.33,均小于2.571,说明两组结果数据没有显著差异,两种方法具有良好的一致性。国标方法是首先酶解肉样品,酶解液用石墨化炭黑柱和氨基柱净化后测定。本方法的定量限低于或等于国标方法的定量限,且国标方法过程繁琐,消耗试剂耗材多,方法测定时间长,因此本方法优于国标方法。

#### 2.5.2 样品测定

采用本方法测定了12个牛肉样品中的50种化合物,这些牛肉均采自某养殖场,饲料中不含人为添加兴奋剂和孕激素。结果见表8,共检出了4种化合物。氢化可的松的检出率最高,含量最高;可的松次之,雄烯二酮和睾酮含量最低。本次检出的氢化可的松和可的松结果与庄玥等^[25]^、杨奕等^[29]^的测定结果基本一致,GB 31650-2019《食品安全国家标准 食品中兽药最大残留限量》规定可的松和氢化可的松属于允许用于食品动物,但不需要制定残留限量的兽药。

**表8:** 12个牛肉样品的测定结果(*n*=2)

Compound	Content range/(μg/kg)	Detection rate/%
Hydrocortisone	3.3-22.6	100
Cortisone	1.5-2.1	67
4-Androstene-3	0.7-1.2	17
Testosterone	0.6-1.5	42

## 3 结论

本研究建立了畜禽肉品中50种激素类药物残留检测方法,该方法采用QuEChERS样品前处理技术,结合超高效液相色谱-串联质谱联用检测方法,回收率和精密度稳定,具有高通量、高灵敏度的优点,能够满足检测需求。该方法可以快速准确地筛查畜禽肉中的44种食源性兴奋剂和6种孕激素药物残留,可用于风险预警、日常监测及应急检测,确保运动员食品食源性兴奋剂含量在安全范围内,该方法的建立可以为重大赛事活动中畜禽肉类食品安全提供有力的技术支撑。
